# Chronic dysphagia caused by Laryngo-vertebral Synostosis after anterior fusion for cervical spine trauma: a case report

**DOI:** 10.1186/s12891-020-3152-5

**Published:** 2020-03-04

**Authors:** Ichiro Okano, Joe Omata, Yushi Hoshino, Yuki Usui, Tomoaki Toyone, Katsunori Inagaki

**Affiliations:** 1 Department of Orthopaedic Surgery, Ohta-Nishinouchi Hospital, 2-5-20 Nishinouchi, Koriyama, Fukushima, 963-8558 Japan; 2 Department of Otolaryngology Head and Neck Surgery, Niigata University Graduate School of Medical and Dental Sciences, Niigata, Japan; 3 Department of Orthopaedic Surgery, Asahi University Hospital, Gifu, Japan; 4 Department of Orthopaedic Surgery, Showa University School of Medicine, Tokyo, Japan

**Keywords:** Synostosis, Dysphagia, Cricoid cartilage, Cervical spine trauma, Anterior fusion, Omohyoid muscle flap

## Abstract

**Background:**

Anterior cervical spine surgery is often associated with postoperative dysphagia, but chronic dysphagia caused by laryngo-vertebral synostosis is extremely rare. We report a case of chronic dysphagia caused by synostosis between the cricoid cartilage and cervical spine after anterior surgery for cervical spine trauma.

**Case presentations:**

We present a case of a 39-year-old man who had sustained complex spine trauma at C5–6 associated with complete spinal cord injury at the age of 22; the patient presented with a 5-year history of chronic dysphagia. Computed tomography demonstrated posterior shift of the esophagus as well as calcification of the cricoid cartilage and its fusion to the right anterior tubercle of the C5 vertebra. A barium swallow study demonstrated significant barium aspiration into the airway and no laryngeal elevation. The patient underwent resection of the bony bridge and omohyoid muscle flap insertion. His symptoms ameliorated after surgery.

**Conclusion:**

Synostosis between the cricoid cartilage and cervical spine may occur associated with cervical spine trauma and causes chronic dysphagia. Resection of the fused part can improve dysphagia caused by this rare condition and omohyoid muscle flap might be a good option to prevent recurrence.

## Background

Anterior cervical spine surgery is often associated with postoperative dysphagia [1]. Most patients with dysphagia improve over time, but a significant proportion have persistent symptoms, with the incidence of chronic dysphagia reported to be 12.5–35% [2–4]. Previous reports suggest that adhesion and protrusion of instrumentation or grafts could potentially cause chronic postoperative dysphagia; however, synostosis of the laryngeal cartilages and cervical spine is extremely rare. We report a case of chronic dysphagia caused by synostosis between the cricoid cartilage and cervical spine after anterior surgery for cervical spine trauma.

## Case presentation

A 39-year-old East Asian man visited our hospital with a 5-year history of progressive dysphagia. At the age of 22, the patient had sustained C5–6 complex fracture/dislocation and complete cervical spinal cord injury at the C7 level due to a motor vehicle accident. No intracranial injury had been recorded. A halo traction was applied on the first day of his hospitalization as a temporary fixation, but definitive treatment was delayed due to severe respiratory distress, which required mechanical ventilation. He underwent anterior C5–6 corpectomy and fusion with iliac crest bone autograft without instrumentation 23 days after the admission. No bone morphologic protein was used. During the initial hospital stay, he underwent tracheostomy because of prolonged respiratory distress due to associated injuries. The tracheostomy site was complicated with methicillin-resistant *Staphylococcus aureus* (MRSA) infection, which was treated with antibiotics and repeated debridement. Since the time of injury, total non-oral nutrition had been continued for over 3 months, because of frequent aspiration and pain during swallowing due to inflammation of the tracheostomy site. No barium swallowing test was performed during the initial hospitalization. After swallowing rehabilitation, the patient could swallow liquid and solid food without aspiration. However, 12 years later, his dysphagia relapsed and gradually progressed. At the time of his 17-year visit, the patient aspirated frequently when he swallowed liquids or solids, to the extent that self-suctioning from the previous tracheostomy site was frequently required.

**Fig. 1:**
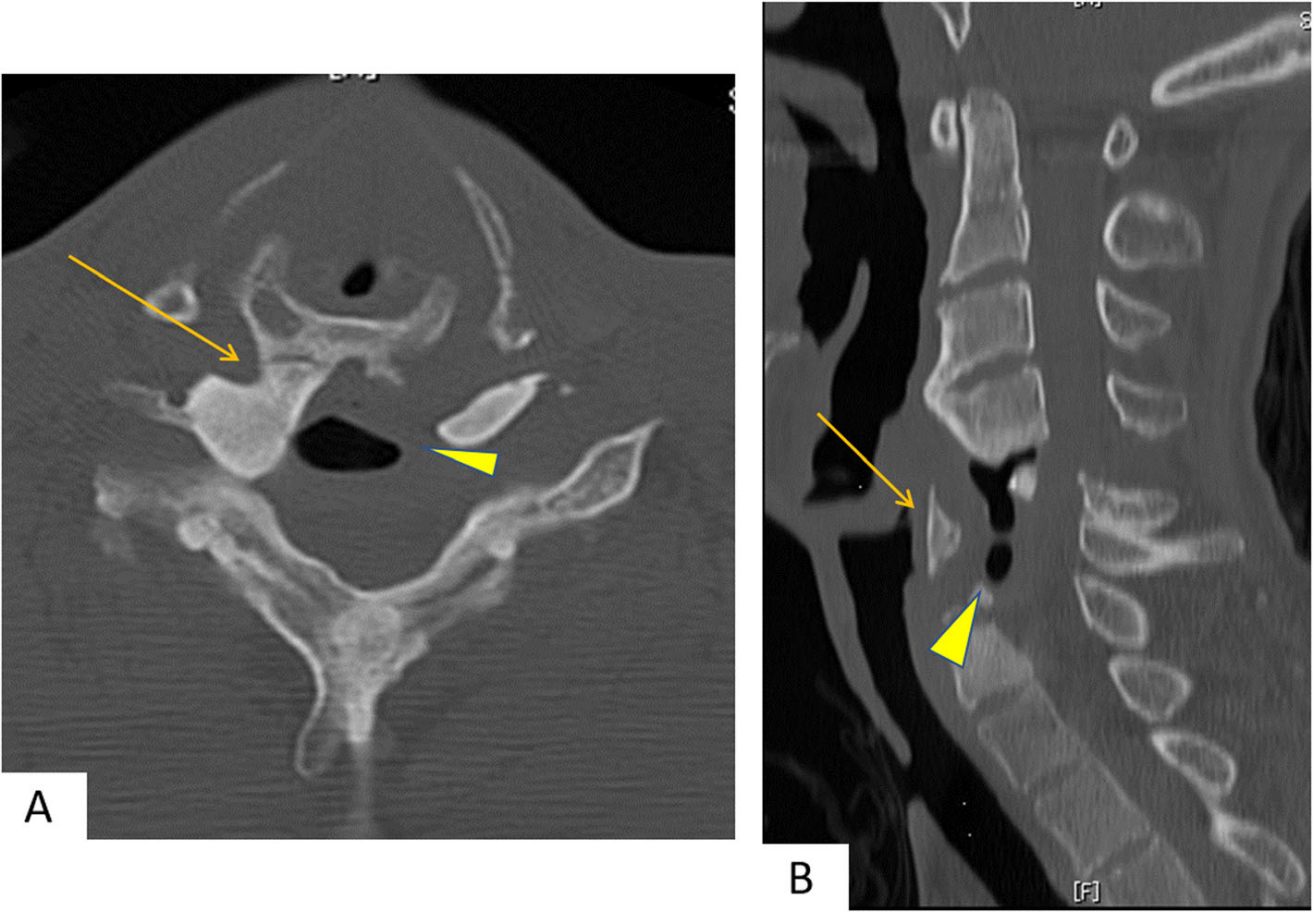
Computed tomography (CT) images at initial examination: **a** An axial image at C5 level showing synostosis between the right posterior part of the cricoid cartilage and the right anterior tubercle of C5 (arrow); total absorption of grafted bone was observed and the esophagus shifted markedly to the posterior side (arrowhead). **b** sagittal reconstruction of the CT showing the posteriorly shifted cricoid cartilage (arrow) and esophagus (arrowhead)

**Fig. 2:**
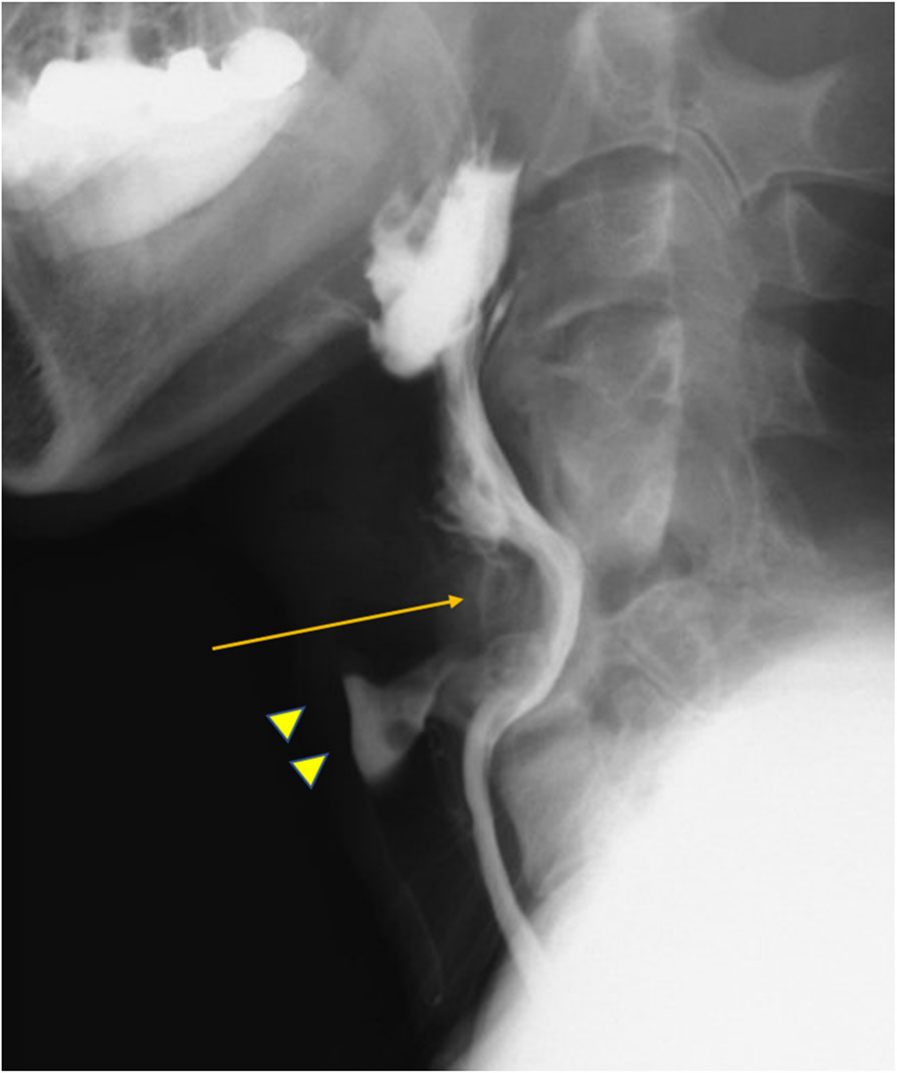
Preoperative barium swallow study: image of early pharyngeal phase showing aspirated barium in the airway (arrowhead). The cricoid (arrow) was in contact with the vertebra. No laryngeal elevation was seen

Computed tomography (CT) scans of the cervical spine revealed almost complete resorption of the bone graft and a posterior shifted esophagus. The injured spinal columns were fused via the posterior and remaining anterior parts of the vertebrae. A bony bridge of heterotopic ossification was observed between the right posterior part of the cricoid cartilage and the right anterior tubercle of the C5 vertebra (Fig. 1). A barium swallow study demonstrated significant barium aspiration into the airway and no laryngeal elevation (Fig. 2) (see Video, Supplemental Digital Content 1).

**Fig. 3:**
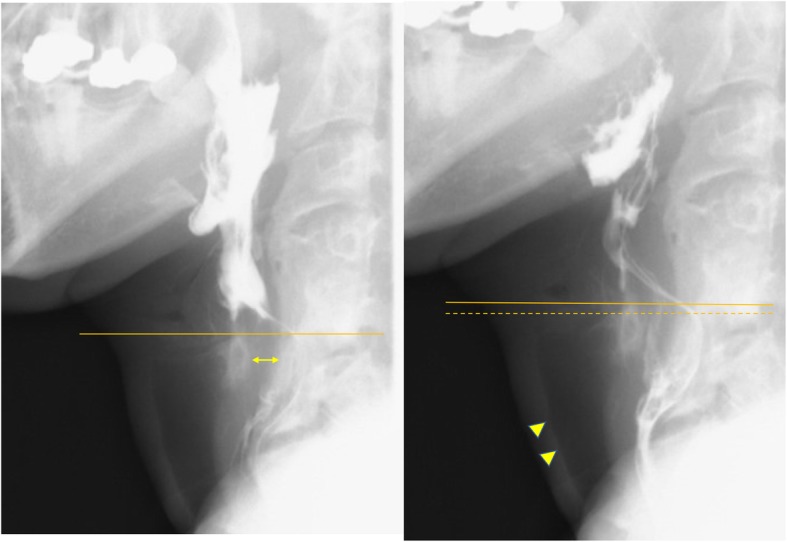
Postoperative barium swallow study on the 10th postoperative day: **a** image of early pharyngeal phase showing interval between the cricoid cartilage and spine (double-headed arrow) (C3/4 anterior osteophyte was also removed). Solid line indicating height of top of the cricoid cartilage. **b** image of late pharyngeal-early esophageal phase showing no aspiration (arrowhead) and improved elevation of the larynges (solid line: top of the cricoid cartilage, dotted line: previous position of top of the cricoid cartilage)

The patient underwent resection of the synostosis; the standard Smith-Peterson approach was utilized through the previous surgical scar. The resection was performed using a high-speed bar and small chisels. The ipsilateral omohyoid muscle (OM) was detached from the hyoid cartilage and the flap was inserted between the vertebral bone and cricoid cartilage to prevent recurrence. A laryngeal suspension procedure [5] was added by otorhinolaryngologists. After surgery, his dysphagia resolved and he could swallow liquid and solids without aspiration. A follow-up barium swallow on the 10th postoperative day demonstrated improved laryngeal elevation and no aspiration (Fig. 3) (see Video, Supplemental Digital Content 2). The patient has had no dysphagia or recurrence 5 years after the surgery.

## Discussion

Laryngeal cartilages are often ossified. Among men, over 35% of thyroid and 20% of cricoid cartilages are ossified [6], but synostoses between laryngeal bony/cartilaginous structures and vertebrae were extremely rare. To the best of our knowledge, this is the first report of postoperative synostosis between the cricoid cartilage and cervical spine.

Among the published literature, we found only two cases of synostosis between the other laryngeal cartilaginous structure, thyroid cartilage, and vertebrae. Han et al. reported a case of synostosis between the thyroid cartilage and C5–7 [7]; their patient showed arrested laryngeal elevation, leading to severe dysphagia like our case. Moses et al. reported a case of posttraumatic synostosis between the thyroid cartilage and C3 [8]. Both cases had cervical spine fractures that were treated conservatively. (7, 8) The summary of previously reported cases is provided in Table 1.

**Table 1:** Summary of previous reported cases with laryngo-vertebral synostosis

Study (year)	Age at the injury	Sex	Injured vertebral level	Synostosis	Treatment for spinal trauma	Interval between trauma and dysphagia	Possible risk factors for synostosis	Treatment for synostosis	Outcome
Moses et al. (1997) [8]	35	Male	N/A (paralysis below C7/8)	Thyroid- C3 right anterior tubercle	Conservative (Halo traction)	20 months	Male sex, spinal cord injury, high-energy trauma	Speech therapy only	Modest improvement: aspiration free, reduced laryngeal elevation, delay in the pharyngeal phase.
Han et al. (2012) [9]	57	Male	C5–7	Thyroid- C5–7 right anterior tubercles	Conservative (neck collar)	7 years	Male sex, bony fragments between fused parts	Surgical resection	Excellent
Okano et al. (presenting)	22	Male	C5–6	Cricoid- C5 right anterior tubercle	Surgical (ACDF)	12 years	Young age, male sex, spinal cord injury, high-energy trauma, fracture-dislocation, prolonged waiting time for surgery, delayed rehabilitation, infection	Surgical resection/ omohyoid muscle flap interposition	Excellent

*N/A* not applicable; *ACDF* anterior cervical decompression and fusion

The pathogenesis of laryngo-spinal synostosis is unclear. In Han’s report, the authors mentioned that small bony fragments were dispersed between the thyroid cartilage and vertebral bones on initial CT; they suggested these fragments might have formed a bony bridge [7]. Posttraumatic synostosis between two neighboring bones has been investigated mainly in the forearm and ankle [9, 10]. The risk factors for posttraumatic synostosis are classified into two main categories: trauma-related and treatment-related [10, 11]. A significant proportion of these risk factors are associated with systemic or local inflammation, which overlap with the general risk factors for posttraumatic heterotopic ossification (HO) (Table 2) [12–14]. Previous studies demonstrated that various inflammatory cytokines were increased in the blood and local tissue among patients with severe HO [15, 16]. In this case, infection around the tracheostomy site and almost total resorption of the grafted bone was observed. According to patient history, it is highly likely that the anterior cervical fusion surgical site was also infected. Another possibility is that the patient might have had an esophageal injury due to the spinal injury itself or ACF, although it was not mentioned in the previous hospitalization record and no workup for esophageal injury was performed. The presence of local inflammation due to surgical site infection or esophageal injury might have contributed to the incidence of cricoid-vertebral synostosis in our case. Moreover, our patient showed almost complete resorption of the grafted bone for possibly associated infection and a posterior shifted esophagus. This anatomical change pushed the laryngeal cartilages closer to the vertebra and might have contributed to synostosis formation, along with other factors mentioned earlier. Lastly, non-oral nutrition had continued for over 3 months in this case. This prolonged immobility of the larynx was one possible reason of synostosis. One interesting difference between laryngo-vertebral synostosis and radioulnar synostosis (tibiofibular synostosis usually does not show any symptoms [9]) were the intervals between the initial injury and synostosis symptom onset. Among patients with laryngo-vertebral synostosis, the intervals between the initial injury and onset of dysphagia were 20 months in Moses’s report [8], over 6 years in Han’s case [7], and 12 years in our case, whereas radioulnar synostosis cases showed earlier symptom onset, typically less than 12 months [10, 17, 18]. This might suggest that laryngo-vertebral synostosis demonstrates more gradual development of bridging bone than synostosis of the forearm, or there might be compensating mechanisms of laryngeal movement, which prevent the synostosis from becoming symptomatic.

**Table 2:** Summary of risk factors for posttraumatic heterotopic ossification and synostosis. Blank fields represent no data/undetermined. N/A: not applicable because of conservative treatment

Factors		Posttraumatic heterotopic ossification [12–16]	Posttraumatic synostosis in the extremities [9–11]		Laryngo-vertebral synostosis [7, 8]	
			Forearm	Ankle	Previous reports	Presenting case
Demographic	Young age (< 30) at the injury	Yes			No	Yes
	Male sex	Yes		Yes	Yes	Yes
	African American race	Yes				No
Trauma-related	Systemic injury severity	Yes				
:Systemic	Head Trauma	Yes	Yes			
	Spinal cord injury	Yes			Yes	Yes
:Local	Extensive soft tissue damage^1^	Yes	Yes			
	High-energy injury mechanism	Yes	Yes^2^	Yes	Yes	Yes
	Fractures in both sides of synostosis at the same level		Yes			
	Fracture-dislocation^3^	Yes	Yes	Yes		Yes
	Comminuted fracture	Yes	Yes			
	Dissemination of bone dust or debris	Yes	Yes		Yes	
	Hematoma formation	Yes				
Surgery-related	Extensive surgical dissection	Yes	Yes		N/A	
	Prolonged waiting time for surgery		Yes		N/A	Yes
	Prolonged immobilization		Yes			
	Delayed rehabilitation		Yes			Yes
	Prominent implant		Yes	Possible^4^	N/A	
	Local infection			Possible^5^		Yes
	Primary bone graft		Yes		N/A	Yes

^1.^ including open fracture, blast injury, and traumatic amputation of the extremities

^2.^ including fracture with syndesmosis injury, which is usually associated with high-energy mechanism

^3.^ including Monteggia fracture in the elbow, and tibiotalar dislocation in the ankle

^4.^ including syndesmotic screw

^5.^ only significant in the univariate analysis

Laryngo-vertebral synostosis likely impairs the dynamic coordinated movement of swallowing. In our case, synostosis resection yielded an excellent result; the follow-up barium swallow study demonstrated improved laryngeal elevation. Han et al. mentioned that laryngeal elevation improved after synostosis resection. Moses et al. treated their patient conservatively, but the reduced laryngeal elevation and delay in the pharyngeal swallowing phase persisted. For severe dysphagia, we believe that synostosis removal should be considered to ameliorate symptoms.

We used the OM flap as an interposition for preventing recurrence. For synostosis in the forearm, although the supporting evidence is limited, various materials are used for preventing recurrence [11]. Those include bone wax, artificial material sheets (silicon or Gore-Tex®), free or pediculed fat flap, and fascia. The OM flap was used for esophageal or pharyngeal perforation associated with anterior cervical spine surgery [19]. Surek et al. reported two cases of esophageal perforation treated with a superior OM flap; the flap can be easily mobilized during neck exploration and the omohyoid is thin, well-vascularized, and of adequate length to reach the mid-to-lower cervical spine [19]. An OM flap might be a good option for interposition after laryngo-vertebral synostosis resection.

## Supplementary information

**Additional file 1.** Supplemental Video Content 1. Preoperative barium swallow study

**Additional file 2.** Supplemental Video Content 2. Follow-up barium swallow study on the 10th postoperative

## Data Availability

Data that support the findings of this study are available from the corresponding author on reasonable request.

## Abbreviations

ACF: Anterior cervical fusion
CT: Computed tomography
HO: Heterotopic ossification
MRSA: Methicillin-resistant *Staphylococcus aureus*
OM: Omohyoid muscle
